# A Hybrid Approach of ANFIS—Artificial Bee Colony Algorithm for Intelligent Modeling and Optimization of Plasma Arc Cutting on Monel™ 400 Alloy

**DOI:** 10.3390/ma14216373

**Published:** 2021-10-25

**Authors:** Mahalingam Siva Kumar, Devaraj Rajamani, Emad Abouel Nasr, Esakki Balasubramanian, Hussein Mohamed, Antonello Astarita

**Affiliations:** 1 Centre for Autonomous System Research, Department of Mechanical Engineering, Vel Tech Rangarajan Dr. Sagunthala R&D Institute of Science and Technology, Chennai 600062, India; drmsivakumar@veltech.edu.in (M.S.K.); esak.bala@gmail.com (E.B.); 2 Department of Industrial Engineering, College of Engineering, King Saud University, Riyadh 11421, Saudi Arabia; eabdelghany@ksu.edu.sa; 3 Department of Mechanical Engineering, Faculty of Engineering, Helwan University, Cairo 11732, Egypt; hussein@h-eng.helwan.edu.eg; 4 Department of Mechanical Engineering, Faculty of Engineering, Ahram Canadian University, Giza 12566, Egypt; 5 Department of Chemical, Materials, and Industrial Production Engineering, University of Naples Federico II, 80138 Naples, Italy; antonello.astarita@unina.it

**Keywords:** modeling, genetic algorithm, adaptive neuro-fuzzy inference system, optimization, artificial bee colony algorithm, box-behnken design

## Abstract

This paper focusses on a hybrid approach based on genetic algorithm (GA) and an adaptive neuro fuzzy inference system (ANFIS) for modeling the correlation between plasma arc cutting (PAC) parameters and the response characteristics of machined Monel 400 alloy sheets. PAC experiments are performed based on box-behnken design methodology by considering cutting speed, gas pressure, arc current, and stand-off distance as input parameters, and surface roughness (Ra), kerf width (kw), and micro hardness (mh) as response characteristics. GA is efficaciously utilized as the training algorithm to optimize the ANFIS parameters. The training, testing errors, and statistical validation parameter results indicated that the ANFIS learned by GA outperforms in the forecasting of PAC responses compared with the results of multiple linear regression models. Besides that, to obtain the optimal combination PAC parameters, multi-response optimization was performed using a trained ANFIS network coupled with an artificial bee colony algorithm (ABC). The superlative responses, such as *R_a_* of 1.5387 µm, *kw* of 1.2034 mm, and *mh* of 176.08, are used to forecast the optimum cutting conditions, such as a cutting speed of 2330.39 mm/min, gas pressure of 3.84 bar, arc current of 45 A, and stand-off distance of 2.01 mm, respectively. Furthermore, the ABC predicted results are validated by conducting confirmatory experiments, and it was found that the error between the predicted and the actual results are lower than 6.38%, indicating the adoptability of the proposed ABC in optimizing real-world complex machining processes.

## 1. Introduction

The current technological revolution has introduced several modern engineering materials, such as alloys and composites, to replace traditional materials in a variety of applications. Monel 400, a Nickel alloy subset, is one of the most widely used materials in engineering and structural applications due to its unique properties, which include a higher weight-to-strength ratio, improved corrosive resistance, and improved thermal properties [1]. A number of secondary processes, such as cutting, milling, drilling, trimming, and so on, are required for effective utilization of these alloys in the aforementioned industries. Due to the increased toughness and lower thermal conductivity of Monel 400 alloys, producing geometrically complex end-use parts using traditional machining processes is more difficult, resulting in frequent tool damage and inferior surface quality [2].

Numerous research studies have recently focused on improving the quality and performance characteristics of conventionally machined Monel 400 alloys by incorporating various accompanying factors during the machining process [3,4,5,6]. Even though previous research has significantly improved the performance of machined parts, achieving the expected part quality for high-precision applications remains difficult. As a result, non-traditional machining processes are required to overcome the shortcomings of traditional machining techniques. For the processing of these difficult-to-machine materials, several researchers have recently focused their attention on numerous non-traditional machining processes such as laser beam machining, abrasive waterjet cutting, electrical discharge machining, ultrasonic machining, and plasma arc cutting processes, etc. [7,8,9,10].

Among these non-traditional machining processes, plasma arc cutting (PAC) is a far-reaching thermal energy-based machining technique commonly used for processing diverse conductive materials to stringent design requirements and intricate geometrical profiles with manifest automation, higher cutting speed, and reasonable cutting cost [11]. Despite its potential benefits, the realization of attractive cutting features such as improving the removal of substrate material, refining the kerf characteristics, and enhancing the surface quality in PAC processed parts are tedious due to the existence of plentiful process related control factors [12]. Due to the abundance of process parameters and complexity in the PAC machining process, it is critical to develop the most precise mapping between the output responses and input parameters for understanding process behavior, parametric investigation, process simulation, and optimization [13]. It also presents an opportunity to understand the shortcomings of the PAC process to attain the requirements of manufacturing industries. In recent years, several researchers have focused on developing comprehensive modeling techniques to predict the machining performance through considering diverse input parameters. Although different statistical approaches have been proposed for modeling the machining processes, such as regression models [14], support vector machines [15], and finite element models [16], soft computing techniques such as fuzzy logic [17], artificial neural networks [18], and adaptive neuro-fuzzy inference system (ANFIS) [19] are prominent in predicting the performance of machining processes because of their progressive computational capability. 

Modeling the advanced machining processes through statistical techniques are tedious due to the existence of complexity and non-linearity in the machining processes, which requires further assumptions and validations and mathematical procedures; this also necessitates a significant amount of experiments. Therefore, these inadequacies restrict the utilization of classical statistical models in real-world industrial applications [20]. On the other hand, due to the capability of model-free estimation, nonlinear mapping, and exceptional learning capability from the experimental data, soft computing techniques can be successfully applied for the intelligent modeling of machining processes. 

Several studies have previously attempted to use soft computing techniques such as genetic programming, fuzzy logic, and artificial neural networks to correlate the relationships between quality and performance characteristics, as well as the input parameters of various machining processes. Fuzzy logic (FL) and artificial neural networks (ANN) have the common ability to solve non-linear and complex engineering problems that are combined with uncertainties and noise [21]. However, ANFIS is a hybrid approach derived from the combinational merits of ANN and FL that includes the exceptional computational capability and supervised learning ability of ANN and the expert knowledge of FL. Combining ANN and fuzzy-set theory can provide benefits and overcome the drawbacks in both techniques. The ANFIS model can be trained without the need for the expert knowledge required for a fuzzy logic model. The ANFIS model benefits from both numerical and linguistic knowledge. ANFIS also makes use of the ANN’s ability to classify data and recognize patterns. The ANFIS model is more transparent to the user than the ANN model and causes fewer memorization errors. As a result, the ANFIS has several advantages, including its adaptability, nonlinear capability, and rapid learning capacity [22]. Recently, ANFIS has been extensively used for the accurate modeling of the input–output relationships of conventional and non-conventional machining processes, such as drilling [23], turning [24], milling [25], electrical discharge machining [26], laser machining [27], and abrasive aqua jet cutting [28], etc., with a high degree of non-linearity. The performance of the ANFIS predictive model is solely dependent on its training parameters (premise and consequent parameters). The ANFIS training parameters that are often explored by trial-and-error methods for non-linear and complex systems, such as advanced machining processes, are tedious and time consuming [29]. In recent years, some researchers have concentrated on improving the learning capabilities of the ANFIS approach by integrating the network with other intelligent tuning techniques. Song and Kasabov [30] have proposed a transductive neuro-fuzzy inference system with weighted data normalization for creating a personalized predictive model. They found that the proposed model can be efficiently utilized for handling large datasets. Benmiloud [31] proposed an improved ANFIS framework for solving non-linear functions. The result of the proposed approach was found to be good, and the approach is well-suited for solving complex problems with minimal error values. Furthermore, several researchers have focused on the classical models and metaheuristic optimization algorithms to optimize the ANFIS training parameters. Because classical approaches may have difficulty regarding local optimal values when training the network, the prediction accuracy declines. However, derivative-free algorithms such as genetic algorithm (GA), simulated annealing (SA), particle swarm optimization (PSO), the cuckoo search algorithm (CSO), and ant and bee colony optimization (ACO and BCO), have been satisfactorily used to deal with this issue during ANFIS training [32,33,34].

Population-based metaheuristic algorithms, particularly GA, are widely used as the training algorithm to construct the most reliable and robust ANFIS networks for complex machining processes through optimizing their premise and consequent parameters by probability-based search strategies. The GA-ANFIS hybrid approach has been extensively used in the modeling of several machining processes such as drilling [35], electrical discharge machining [36], and milling [37,38]. However, the modeling of PAC parameters using a GA-ANFIS integrated approach has not been dealt with in the literature.

Apart from these extensive research efforts in the intelligent modelling of machining processes, various researchers from around the world have focused on a variety of traditional and contemporary metaheuristic optimization techniques to optimize process parameters. Traditional optimization techniques often produce discrete combinations of a predetermined level of process parameters, and these combinations are not always optimal [39]. Due to there being less computational effort and with the prospect of searching for a large set of feasible solutions, metaheuristic optimization techniques such as GA, SA, PSO, teaching-learning based optimization (TLBO), etc., are often efficiently utilized for the optimization of non-linear processes such as machining and additive manufacturing. Because PAC is a highly non-linear and complex machining process governed by numerous processing parameters, a small deviation in process parameter can drastically affect the quality and performance characteristics of machined components. Recently, some researchers have utilized metaheuristic algorithms such as GA [40,41,42] and the whale optimization algorithm [43] for effective optimization of PAC processes and their characteristics. However, the artificial bee colony algorithm (ABC) has not been utilized previously to perform optimization studies on ABC processes. The main advantages of the ABC algorithm over other optimization methods for solving optimization problems are its simplicity, high flexibility, and robustness, and the fact that it has few control parameters, can be easily combined with other methods, has the ability to handle objectives with a stochastic nature, has fast convergence, and can be used for both exploration and exploitation [44].

Therefore, the present work focuses on developing a soft computing approach for process modeling and optimization of PAC processes. The approach used consists of two elements: firstly, an integrated expert system of GA-ANFIS is proposed for the effective assessment of PAC performances, including surface roughness (*Ra*), kerf width (*kw*), and micro hardness (*mh*) by considering cutting speed, gas pressure, arc current, and stand-off distance as the independent variables. GA is effectively utilized as a training algorithm for the ANFIS network to estimate optimal network modeling parameters. The effectiveness of the proposed GA-ANFIS approach is evaluated using various statistical elements such as mean absolute percentage error and root-mean square error. Secondly, multi-objective optimization is performed to identify the optimal parameters for improving the performance characteristics of PAC processes using an artificial bee colony algorithm. 

## 2. Proposed Methodology

### 2.1. Response Surface Methodology

**Figure 1:**
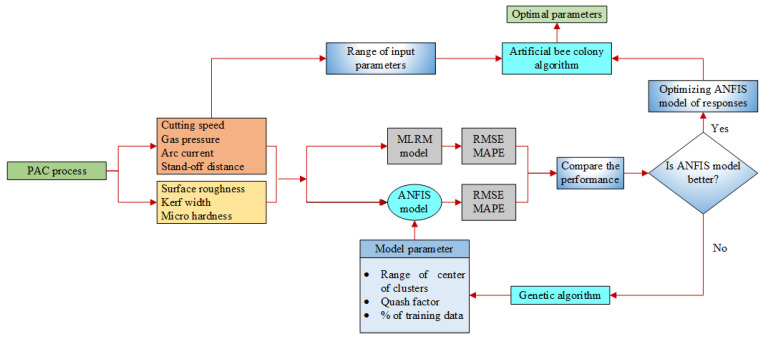
Flow chart for proposed methodology.

A creative experimental approach can significantly reduce the number of experimental trials while maintaining the precision of any manufacturing process. As a result, the current work employs a box-behnken design (BBD) based on response surface methodology (RSM), a statistical and cost-effective design of experiment approach, to design and conduct the PAC experiments [45]. Thirty experiments were designed and performed with six replicates in a block with four-factors and three levels. The second-order polynomial relation that evolved through RSM was utilized to express the behavior of the PAC process, which is given by:

(1) $$Y=\beta_{0}+\sum_{i=1}^{n}\beta_{i}X_{n}+\sum_{i=1}^{n}d_{i}X_{i}^{h}\pm \varepsilon$$

where *h* is the degree of the polynomial (i.e., 2 for the present investigation), *β* represents the coefficients of regression, and *X* and *Y* are considered as response and predictor variables, respectively, while *ε* indicates the normal distribution. The empirical models were developed from experimental analysis data. The graphical representation of the proposed research is depicted in Figure 1.

### 2.2. Adaptive Neuro-Fuzzy Inference System

The ANFIS is a hybrid predictive approach that combines artificial neural networks and fuzzy logic systems to map the relationship between uncertain input and output variables [46]. The ANFIS model was created by combining the network topology of a fuzzy system with the back-propagation algorithm of a neural network to reduce the optimization search space and automate the fuzzy system’s parametric training. The training error will be reduced as a result of the ANFIS model, while the learning and optimization capabilities will be enhanced.

**Figure 2:**
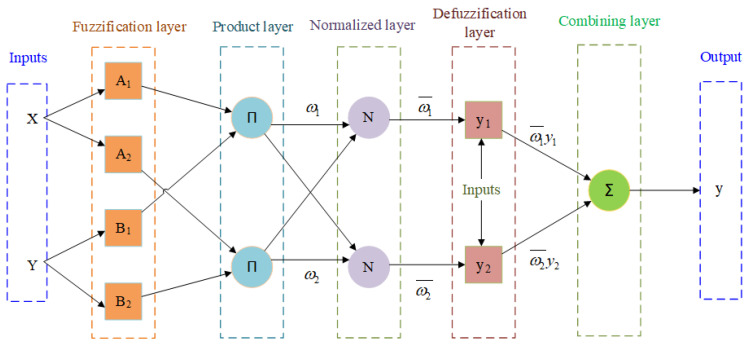
ANFIS framework.

The proposed ANFIS architecture consists of five layers, as shown in Figure 2. They are: (1) input fuzzification layer, (2) product layer, (3) fuzzy rule base construction or normalized layer, (4) de-fuzzification layer, and (5) output layer. The description of the relation between the input and output of each layer in ANFIS is discussed below:

First layer (Input fuzzification layer): In this layer, crisp inputs (*A*_1_, *A*_2_ and *B*_1_, *B*_2_, the nodes, and m and n, inputs of the nodes) are transforms to linguistic terms ($\mu A_{i}$, $\mu B_{j}$) using the membership functions. The output of this layer can be expressed as:

(2) $$\begin{array}{l} O_{1,i}=\mu A_{i}\left(m\right),i=1,2\\ O_{1,j}=\mu B_{j}\left(n\right),j=1,2 \end{array}$$

Here, $A_{i}$ and $B_{j}$ represents the linguistic labels of inputs; m and n are input variables to node i,j, and $O_{1,i}$; and $O_{1,j}$ denotes the output functions. The variables on this layer are referred to as premise parameters. 

Second Layer (Product layer): Every node in this layer is a node labelled II, which multiplies all the input signals and sends it to its output. The outputs $\omega_{1}$ and $\omega_{2}$ of this layer are the weight functions of the next layer. The output node function of this layer can be written as:

(3) $$O_{2,i}=\omega_{i}=\mu A_{i}\left(m\right)\cdot \mu B_{j}\left(n\right),i=1,2$$

Here, $O_{2,i}$ denote the output of the product layer. Each node output of this layer represents the reasoning capability of one fuzzy logic rule.

Third Layer (Fuzzy rule base construction or normalized layer): In this layer, the nodes are labelled as N and a series of fuzzy logic rules are built in advance to express the behavior of the prediction process. The *i*th node calculates the ratio of the *i*th rules firing strength to the sum of all other firing strengths, given as follows:

(4) $$O_{3,i}=\bar{\omega_{i}}=\frac{\omega_{i}}{\omega_{1}+\omega_{2}},i=1,2$$

where,$O_{3,i}$ denote the output of the normalized layer and $\bar{w_{i}}$ denotes the normalized firing strength. 

Fourth Layer (De-fuzzification layer): This is the last part of fuzzy rule, whose nodes are adaptive. In this layer, the Takagi–Sugeno fuzzy type rule (IF–THEN) is applied in the weighted output in each node. The de-fuzzy relationship between the input and output of this layer can be written as:

(5) $$O_{4,i}=\bar{\omega_{i}}f_{i}\left(p_{i}m+q_{i}n+r_{i}\right),i=1,2$$

Here, $O_{4,i}$ denotes the output of the fourth layer and $p_{i}m$, $q_{i}n$, and $r_{i}$ denote the linear or consequent parameter of the node.

Fifth Layer (Combined output layer): This is the overall output layer, which consists of two nodes whose nodes are labelled as ∑. The output of this layer is the total of the input signals, where the first node represents the results of kerf deviation and the second node represents the results of the material removal rate. The results of this layer can be written as:

(6) $$O_{5,i}=\sum_{i}\bar{\omega_{i}}f_{i}=\frac{\sum_{i}\omega_{i}f_{i}}{\sum_{i}\omega_{i}},i=1,2$$

where $O_{5,i}$ represents the output of the fifth layer. 

The mathematical form of membership functions that are used in the fuzzy rule is summarized as follows:

(7) $$\mu (X,a,b,c)=\exp\left[-{\left(\frac{x-c}{a}\right)}^{2}\right]$$

where a and b vary the width of the curve and c locates at the center of the curve. The parameter c should be positive, and all these parameters are called the premise parameters. 

#### Training of ANFIS Network Using the Genetic Algorithm

**Figure 3:**
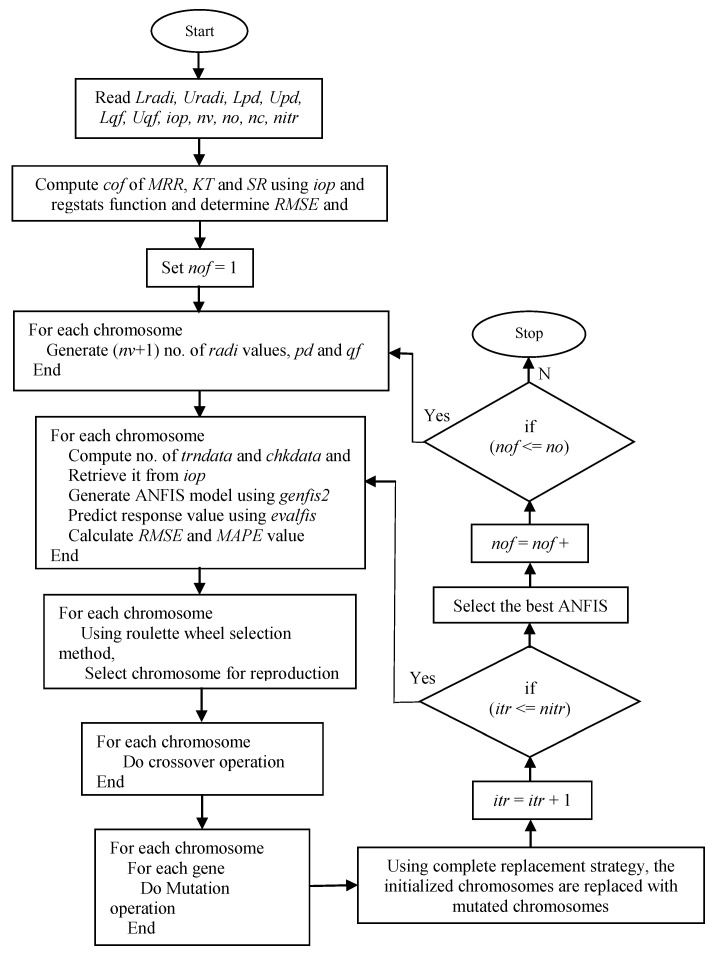
Implementation of GA for the development of ANFIS model.

Deciding the parameters of the ANFIS prediction model, such as clustering radius, quash factor, and percentage of training data, is difficult because most of these parameters are selected from the knowledge of the users and/or by using a trial-and-error approach. To overcome this problem in the ANFIS training of the genetic algorithm, an efficient metaheuristic optimization tool is employed to automate the process of deciding these parameters and to improve the learning rate through minimizing the prediction error. The process of training the ANFIS model using GA is presented in Figure 3. The root-mean square error (RMSE) and the mean absolute percentage error (MAPE) act as the objective functions of the ANFIS-GA training algorithm. These two approaches are hybridized to obtain the benefits of ANFIS and GA so that the proposed model can perform more efficiently.

GA is a well-known population-based evolutionary optimization algorithm that, in contrast with conventional optimization techniques, generates global optimum solutions for constrained and unconstrained problems with stochastic, non-linear, and non-differentiable objective functions using natural selection principles [47]. Many researchers have suggested that global optimization techniques such as genetic algorithms, particle swarm optimization, and artificial bee colony algorithms might prevent fuzzy logic, ANN, and ANFIS from falling into a local minimum [48,49,50,51,52,53,54]. 

By altering the set of randomly generated initial populations in GA, a better solution can be predicted. Each initial population’s fitness is assessed, with a higher fitness indicating that the solution is good. New solutions are generated in each iteration using reproduction, crossover, and mutation functions to operate on the obtained population. The obtained solutions are evaluated and tested for the termination criterion based on the essence of GA after several iterations. Individuals known as good solutions are chosen by reproduction operators and subsidize the population in the following generation. To generate the next solution, crossover operators combine chromosomes based on their likelihood of crossing. The chromosomes are primed by the mutation operator to change and adjust their values, which aids in population diversity. These steps are repeated until the termination criterion is satisfied, or a chromosome has achieved the best fitness and thus is considered the best solution for the objective function. Initially, the parameters of genfis2 have been given as the input for GA as the binary string, and the network parameters are represented by the genes of individual chromosomes. The best solutions are then obtained through generating the initial random parameters by means of the population of strings (i.e., *genes* *→* *chromosomes* *→ population → generation*). In this study, the chromosomes for the next generation are chosen using a roulette wheel. Furthermore, the genes of each reproduced chromosome were subjected to crossover and mutation operations. Finally, the values of fitness functions are obtained by replacing the initialized chromosomes with mutated chromosomes using a complete replacement strategy.

### 2.3. Artificial Bee Colony Algorithm

The artificial bee colony (ABC) algorithm is a population-based metaheuristic technique that mimics the foraging behavior of honeybees to explore global solutions for intricate real-world optimization problems [55]. In the ABC algorithm, the exchange of information among the bees in dancing areas are employed through three categories of bee: employed, unemployed, or onlooker and scout bees. Among these, the employed bees are currently engaged in exploiting the food sources (solution of a problem), the unemployed or onlooker bees continuously look out for a food source to exploit, while the scout bees are waiting in the nest and are establishing the food source through information shared by employed bees (exploration). In the ABC algorithm, the food source is confined within D-dimensional space and signifies a feasible solution for the optimization problem. The quantity of nectar in the food source improves the probability of attracting the onlooker bees, which is considered as the fitness function for the optimization problem. The algorithm is controlled by three major parameters, namely colony size (number of bees), limit (number of trials), and the maximum cycle. 

The implementation of the ABC algorithm consists of four major steps, including initializing the position of food sources, colony size, and algorithm variables; searching for the position of new food sources by employed bees; searching the position of new food sources by unemployed bees; and the scout bee phase. Stepwise implementation of these phases is explained, as follows [56]:

*Step* *1* *Initialization of Bee colony*

Forty random sets of values are generated using Equation (8) between the lower ($L_{j}$) and upper ($U_{j}$) boundary values of the parameters cutting speed, gas pressure, arc current, and stand-off distance and this is considered as the initial position ($P_{ij}$) of 40 bees. The index for number of bee and its dimensional position, i.e., number of parameters, are considered as *i* and *j*.

(8) $$P_{ij}=L_{j}+R_{ij}\left(U_{j}-L_{j}\right)$$

*Step* *2* *Evaluation of fitness value of bee*

For each bee, the response value of *Ra*, *kw*, and *mh* are calculated using the ANFIS model developed in the previous stage for the experimental values. Due to multiple objectives with contradictory natures (both minimization and maximization), using the Technique for Order of Preference by Similarity to Ideal Solution (TOPSIS) method, it is converted into a single objective and the same is considered as the fitness value of each bee.

*Step* *3* *Selection of employed bees and unemployed bees*

The bees are arranged in descending order of TOPSIS values, and the first 20 bees are selected as employed bees to carry out the further process in the algorithm. The remaining 20 bees are considered as unemployed bees. The positions, i.e., parameter values, along with this response values corresponding to first rank bees, are stored in a separate file and considered as the result of the first iteration.

*Step* *4* *Determination of new position of employed bees*

The new position (*Q_ij_*) of employed bees is calculated using the following Equation (9), where *δ_ij_* represents a constant between −1 and 1 and *k* represents a random number within the maximum number of employed bees. These values are checked against its boundary values and then the response values are calculated using the ANFIS Model. Using TOPSIS, the best 20 bees are selected by combining the old and new response values.

(9) $$Q_{ij}=P_{ij}+\delta_{ij}\left(P_{ij}-P_{kj}\right)$$

*Step* *5* *Determination of new position of unemployed bees*

Based on the fitness value of unemployed bees, the probability and cumulative probability are calculated using Equations (10) and (11). The onlooker bees are selected to look for new food sources using the roulette wheel selection method. The new positions are calculated using Equation (9), and the fitness values are determined as discussed in step (2). An individual onlooker bee’s fitness value is compared, corresponding to its old and new position. If the old position’s fitness is good compared to the new position’s fitness, then the onlooker bee is noticed. If the noticed count of the onlooker bee exceeds the number 10, i.e., 50% of unemployed bees, then the scout bee is initiated, with the position calculated by Equation (8), and its fitness value is then computed as per the procedure given in the evaluation of fitness value of the bee.

(10) $$r_{i}=\frac{f_{i}}{\sum_{i=1}^{nb}f_{i}}$$

(11) $$c_{i}=\sum_{l=1}^{i}f_{l}$$

*Step* *6* *Replacement of initial population of bee*

The new position of both employed and unemployed bees are replaced with the initial population of the bee’s location, along with its response values. Using the TOPSIS (Technique for Order of Preference by Similarity to Ideal Solution) method, the fitness values are calculated for the new positions of the bees.

*Step* *7* *Stopping criteria*

**Figure 4:**
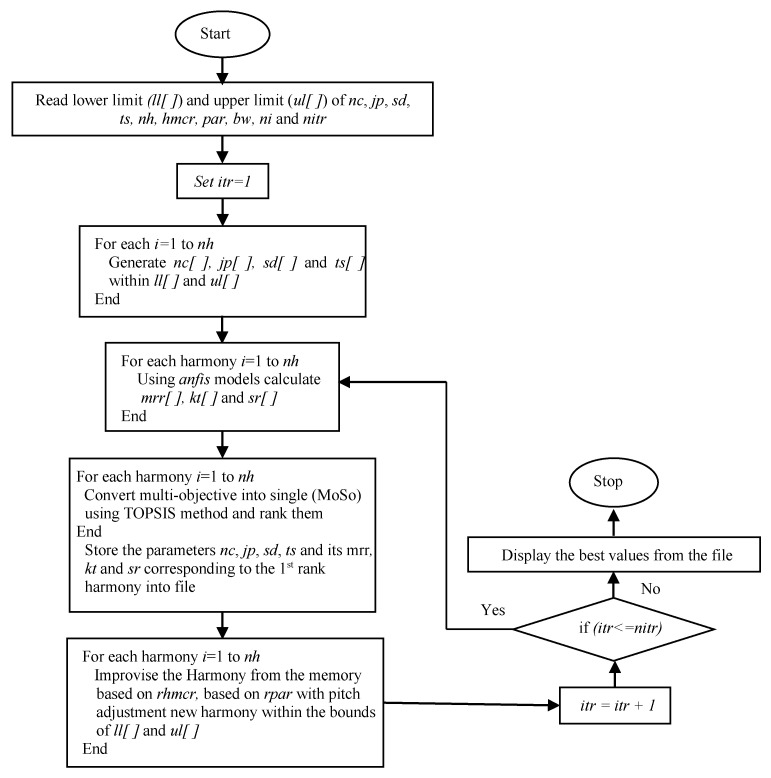
Implementation of the ABC algorithm for multi-response optimization.

The above steps, starting from *c* to *f*, are repeated until the stopping criteria is reached, i.e., up to 50 iterations. The step-by-step implementation of the ABC algorithm for solving the present multi-response PAC problem is presented in Figure 4.

## 3. Plasma Arc Cutting Experiments

**Figure 5:**
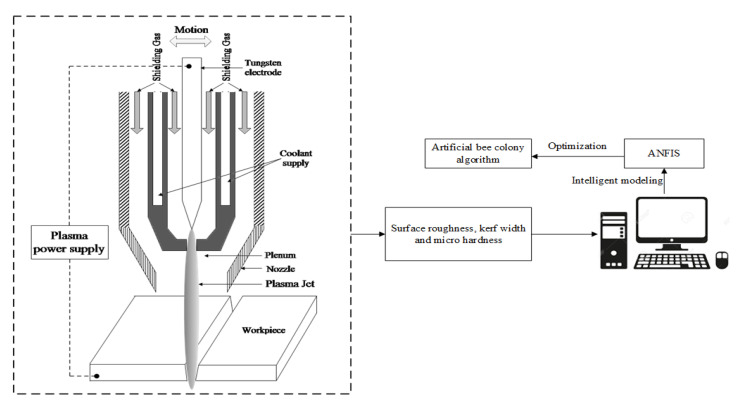
Schematic representation of the experimental set-up.

Experimental investigations on PAC of Monel 400 alloy, with the material composition of 63% nickel, 31.6% copper, 2.5% steel, 2% manganese, 0.5% silicon, and 0.3% carbon, was performed as per the experimental design. Specimens of 3 mm thickness with 200 mm width and 200 mm length were considered as the workpiece material. The PAC experiments were performed using an industrial purpose plasma arc cutting system (Pro arc CNC profile cutting system, Pro-arc welding and cutting systems private limited, Pune, India). The schematic of the PAC experimental system is presented in Figure 5. The PAC setup was furnished with PlasmaCAM CNC software to confirm the accurate motion of the plasma jet through the nozzle. Compressed air was used as a shield gas to generate high-energy plasma to thaw out and spew the smelted metal onto the substrate surface. The precision in cutting operation was accomplished through a servo-operated torch comprising a copper nozzle with an air-cooled swirl. 

The PAC of a 25 mm length was performed in each experimental run along the width of the specimen to appraise the surface roughness (*Ra*), kerf width (*kw*), and micro hardness (*mh*). Four significant material and process related parameters, such as cutting speed (A), gas pressure (B), arc current (C), and stand-off distance (D), were used to regulate the PAC experiments and to assess the selected response characteristics. The levels of these parameters were finalized through conducting exhaustive preliminary experiments by changing one variable at a time. The numerical values of the selected parameters and their levels are presented in Table 1. 

The surface roughness of the kerf cut area was measured using a Universal 3D Profilometer (Rtec instruments, San Jose, CA, USA), and an average of three measurements was used in order to eliminate statistical errors. Similarly, the kerf width of the top cut surface was measured with the aid of a high precision optical microscope (RTM 900, Radical Scientific Equipments Private Limited, Ambala, Haryana, India) at 20× magnification. Micro hardness values were analyzed for evaluating the impact of the thermal effect on the sub-surface of the cutting zone using a Wolpert-micro-Vickers hardness tester (402 MVD, Wilson Instruments, Lake Bluff, IL, USA) with a load of 300 g and a dwell time of 10 s. The experimentally measured responses are presented in Table 2. 

## 4. Result and Discussions

### 4.1. Estimation of PAC Characteristics by GA Tuned ANFIS Model

The present study utilizes GA as a training tool for predicting optimal ANFIS parameters. The selection of premise parameters (parameters related to membership functions) and consequent parameters (parameters related to the defuzzification process) of ANFIS is difficult because most of these parameters are selected from the knowledge of users and/or a trial-and-error approach. To overcome this issue in ANFIS, an efficient metaheuristic optimization tool, GA, is employed to automate the process, which can also improve the learning rate through minimizing the prediction error. The hybridization of ANFIS-GA, consists of three major stages, which include designing the model, training the network, and evaluating the trained model. These two approaches are hybridized to obtain the benefits of ANFIS and GA so that the proposed model can perform more efficiently. 

In this work, the ‘genfis2’ MATLAB™ function, sugeno-type subtractive clustering method is used to generate a FIS model. The behavior of the subtractive clustering ANFIS model is varied based on the RADII, quash factor, accept ratio, and rejection ratio. The range of influence of the cluster center for each parameter and response is defined as RADII and it falls between 0 and 1. Usually, smaller cluster RADII will yield good results; hence, in this work, it is assumed to be between 0.13 and 0.5. The neighborhood cluster center is determined by multiplying the RADII with the quash factor. Apart from the above factors, the amount of data available for checking and testing also influences the performance of the FIS model. Generally, both accept and reject ratios are fixed as default value of 0.5 and 0.15, respectively. In developing the best FIS model for each response, the other factors, such as RADII, quash factor, and amount of training and checking data are considered as variables.

In the first step of GA-ANFIS modeling, the control factors of the PAC process, i.e., cutting speed, gas pressure, arc current, and stand-off distance, are set as input factors, and *Ra*, *kw*, and *mh* are considered as output factors. The FIS parameters, such as cluster radius, quash factor, and percentage of data required to train the network, are then selected in order to enhance the accuracy of the trained network with minimal prediction errors. Table 3 provides the range of ANFIS parameters selected for initializing the network training using GA. The fuzzy rules are developed by clustering the selected process parameters into several values and combining two or more membership functions. Due to the existence of several process parameters, there is a need for the development of considerable membership functions to establish the rule-based relationship between input parameters and selected response factors. Therefore, in this work, the subtractive fuzzy clustering approach [57] is incorporated. Among the several membership functions (MF), a gaussian shaped MF is selected because of its smoothness and concise notation in forecasting the responses. 

To develop the GA-ANFIS hybrid approach, the optimal parameters of GA were selected by performing several parametric studies. A program was coded in the MATLAB environment (Version: Matlab 2020b™, System configurations: 8 GB RAM, 1 TB Hard disk, Intel Core i5 processer and off-line system) to develop the GA-ANFIS model. The ANFIS network was trained by optimizing its premise and consequent parameters to own the closer relationship between input and output variables using GA. The key parameters used in GA for the optimization studies of the ANFIS network are given in Table 4. These parameters were obtained through performing several iterations in the trial-and-error method by keeping the references of existing studies [58].

**Figure 6:**
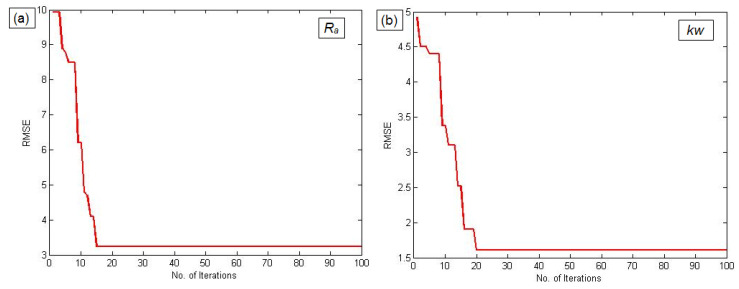

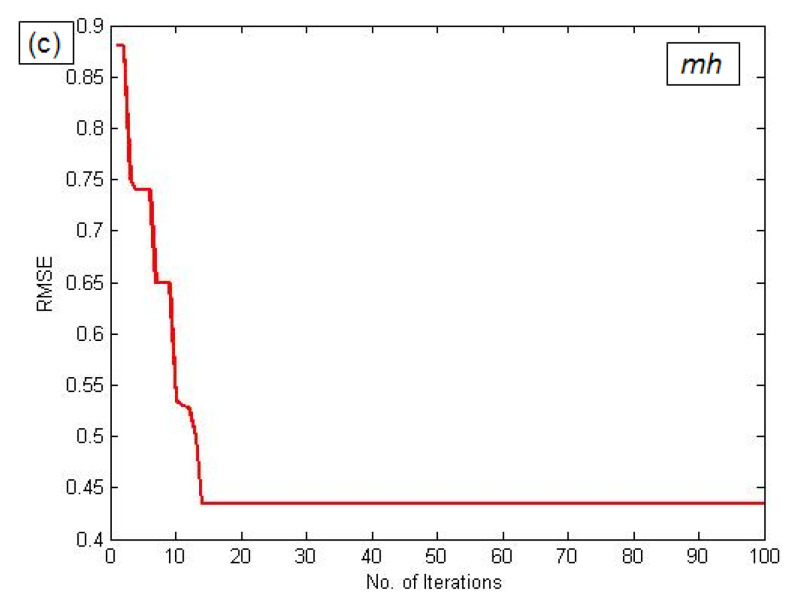
GA convergence plots for ANFIS parameter tuning: (**a**) *Ra*, (**b**) *kw,* and (**c**) *mh*.

The convergence plots of GA shown in Figure 6a–c exhibit the correlation between the number of iterations and their significance on RMSE for selected performance measures such as *Ra*, *kw*, and *mh*. 

**Figure 7:**
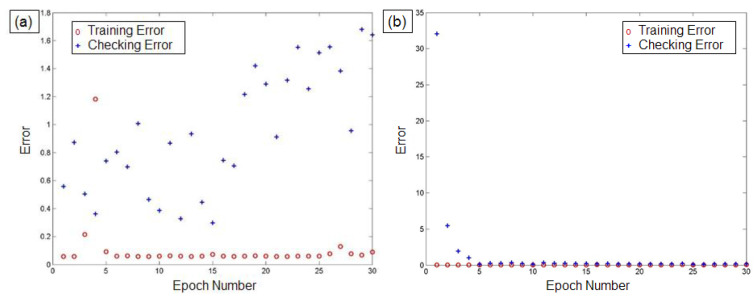

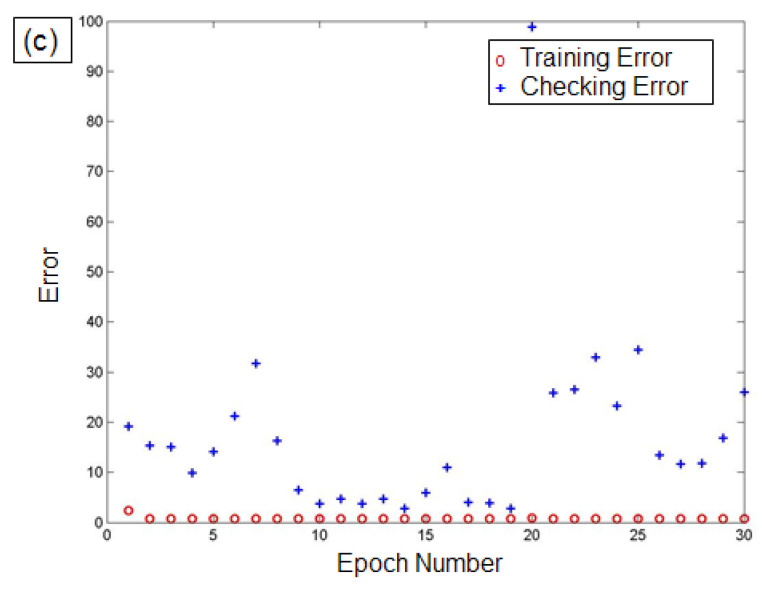
ANFIS training and checking errors for: (**a**) *Ra*, (**b**) *kw*, and (**c**) *mh*.

From the results of GA trained ANFIS, the optimal parameters of the FIS network were obtained. As per the available twenty-nine items of experimental data, twenty datasets were considered as the optimal values for the training of the ANFIS network. The remaining nine data items were considered for validating the performance of the trained network. Figure 7a–c shows the scatter plots of the training and checking errors obtained from ANFIS. The errors of the training and testing data of actual and forecast values for *Ra*, *kw*, and *mh* are listed in Table 5. From these results, it is observed that the proposed GA trained ANFIS model provides a close correlation between the PAC parameters and their responses, with minimal training and testing errors, which demonstrates the reliability of the proposed approach.

### 4.2. Evaluation of GA-ANFIS Prediction Models through Statistical Analysis

In this work, a GA-ANFIS hybrid model was proposed to establish the relationship between the PAC parameters on the quality and performance indices, such as *Ra*, *kw*, and *mh*, of machined parts. Statistical analysis was performed to evaluate the efficacy of the proposed GA-ANFIS model. For comparison purpose, multiple linear regression models (MLRM), which are a widely used tool to obtain the best-fit mathematical equation when there is more than one predictor variable, were developed for each response. The same datasets were used in constructing the GA-ANFIS model. The precision of the proposed hybrid model was assessed by calculating the root-mean square error (RMSE) and mean absolute percentage error (MAPE) values. The RMSE and MAPE were calculated using the following relations:

(12) $$\mathrm{RMSE}=\sqrt{\frac{1}{M}{\sum_{Z=1}^{M}\left(S_{Z}-Y_{Z}\right)}^{2}}\times 100$$

(13) $$\mathrm{MAPE}=\frac{1}{M}\sum_{z=1}^{M}\left|\frac{S_{z}-Y_{z}}{S_{z}}\right|\times 100$$

where M is the total number of the training sample, $S_{Z}$ is the real output value, and $Y_{Z}$ is the ANFIS output value in training. 

**Figure 8:**
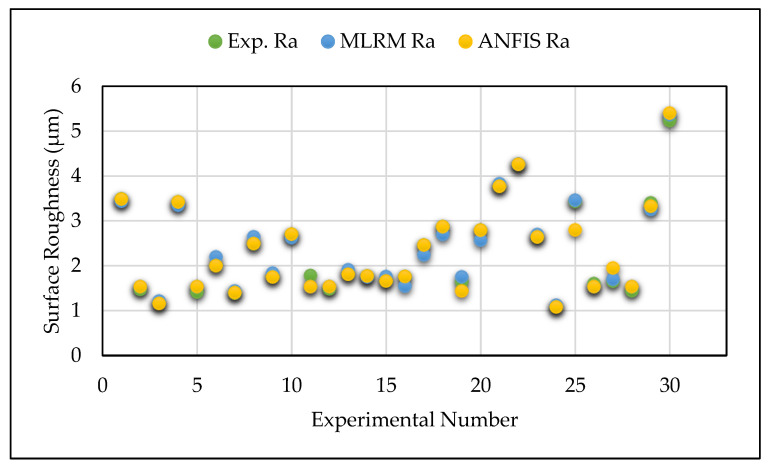
Comparison of ANFIS and MLRM predicted vs. experimentally measured surface roughness values.

**Figure 9:**
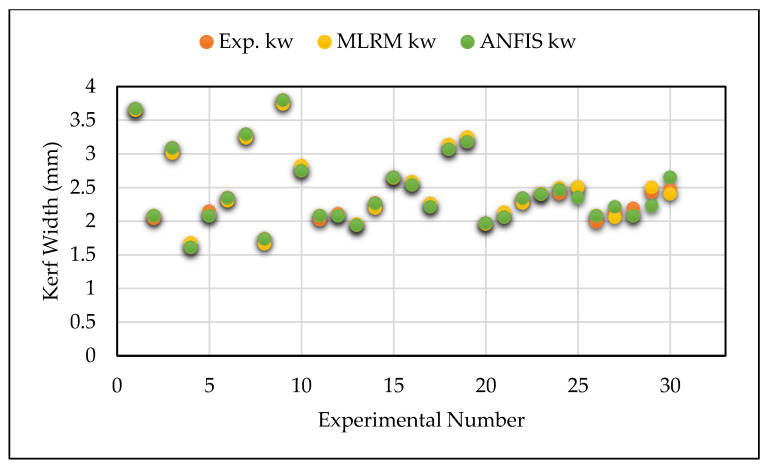
Comparison of ANFIS and MLRM predicted vs. experimentally measured kerf width values.

**Figure 10:**
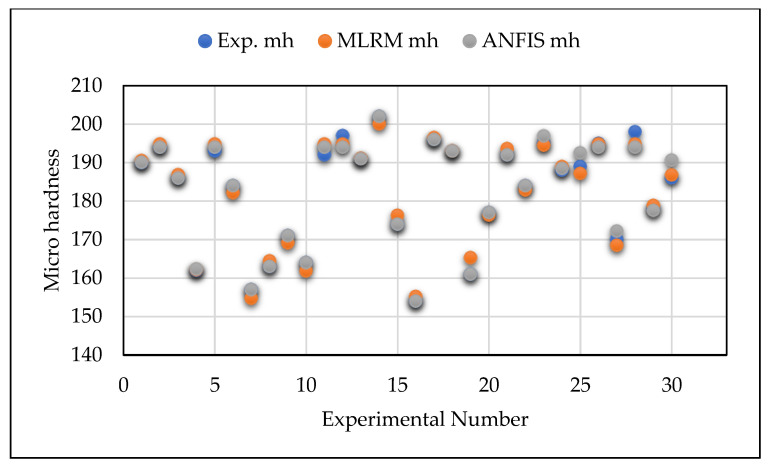
Comparison of ANFIS and MLRM predicted vs. experimentally measured micro hardness values.

The lower values of RMSE and MAPE indicate the higher accuracy and minimal error of the predictive model. Table 6 reports the results of these criteria in the present investigation. Based on the information specified in Table 6, it is thought that the predicted RMSE and MAPE values of GA-ANFIS were superior compared to the values obtained from MLRM. Figure 8, Figure 9 and Figure 10 show the comparative analysis of the predicted values of GA-ANFIS and MLRM, comparing them with the experimentally measured responses. It is inferred that the predicted and experimental measured response values are close to each other. However, the PAC responses predicted by GA-ANFIS are closer to the experimental measured data, compared to the MLRM outcomes, i.e., the error is very small. From these measures, it can be inferred that the GA-ANFIS hybrid approach empowers progressively effective and precise estimation of the PAC process. Moreover, the computational time for conventional and parametric tuned ANFIS has been evaluated in order to estimate the computational complexity of the proposed approach. It was found that the parametric tuned ANFIS took 14.6 s to complete the simulations for minimizing the RMSE and MAPE values. On the other hand, the conventional ANFIS took only 2.3 s for each iteration (trial) to obtain the training network. However, the conventional ANFIS required numerous trials in order to obtain minimized error values, and this generally consumes more computational time and effort. Therefore, the systematic approach of GA tuned ANFIS has been proven as an effective approach for the modelling of complex problems. 

### 4.3. Influence of PAC Parameters on Selected Responses

**Figure 11:**
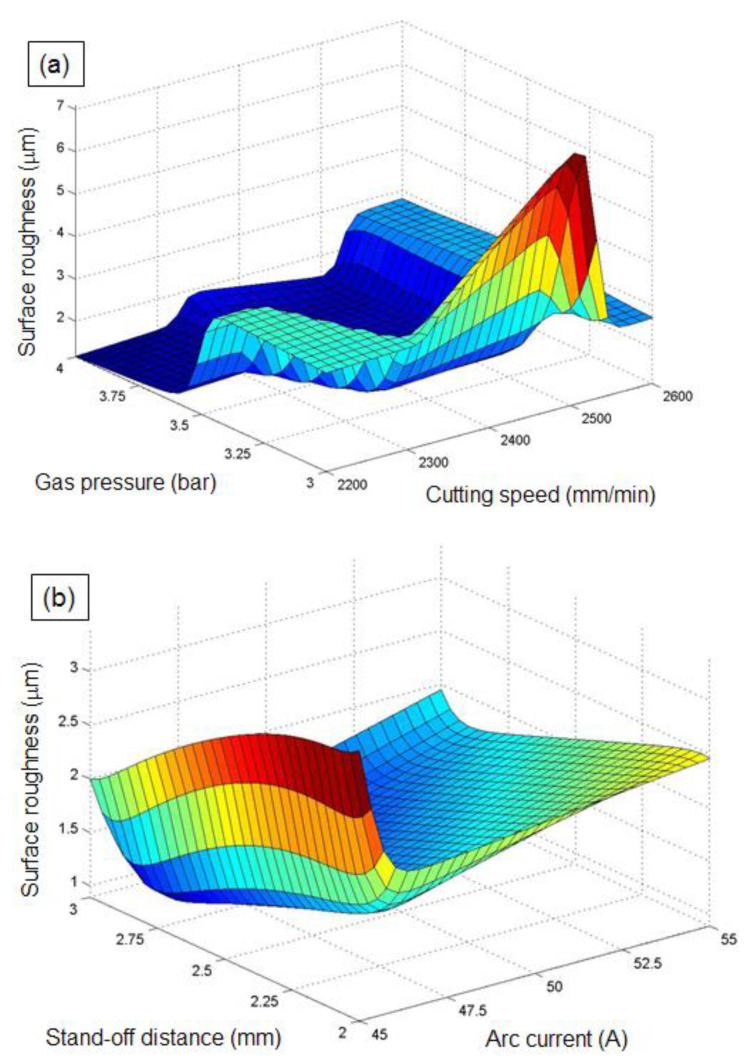
Influence of PAC parameters on *Ra:* (**a**) influence of cutting speed and gas pressure on *Ra*, and (**b**) influence of arc current and stand-off distance on *Ra*.

The influence of PAC parameters on the selected response characteristics were evaluated with the aid of ANFIS three-dimensional surface plots. From the surface plots, two parameters were varied for investigation, while the other two parameter values were kept at middle levels. The impact of PAC control factors on *Ra* is described with the assistance of three-dimensional plots (Figure 11a,b). The interaction dominance of CS and GP on the *Ra* is depicted in Figure 11a. The plot indicates that the *Ra* is increasing with the increase in CS from 2200 mm/min to 2600 mm/min, whereas the *Ra* is linearly decreasing with the increase in GP. As CS increases, the arc coherence of the plasma will be deviated from its axis, resulting in an enlarged kerf and simultaneous reduction in surface quality [59]. Therefore, the *Ra* increases with an increasing of CS. It can be seen that the higher GP results in a smooth cut surface with minimized roughness values. As the GP increases, the melted substrate materials are eventually ejected from the cut surface, and hence the quality of the kerf zone will be improved. Moreover, an improved surface quality with an *Ra* of 4.01 µm is observed at a higher GP (4 bar) and lower CS (2200 mm/min).

Figure 11b shows the effect of AC and SOD on the *Ra*. As can be seen from the interaction plot, the increase in AC slightly increases the Ra, whereas the augmentation of SOD within the selected range is found to have an insignificant impact on the surface quality. The maximized AC leads an erratic arc to melt and evaporate the substrate from the cutting zone, and hence the unpredicted material removal will happen at the surroundings of the actual cutting zone during higher AC, which leads to an augmented oxidation zone and *Ra* [60]. An improved surface quality (*Ra* of 1.89 µm) is attained by a combination of lower AC (45 A) and medium SOD (2.5 mm). 

**Figure 12:**
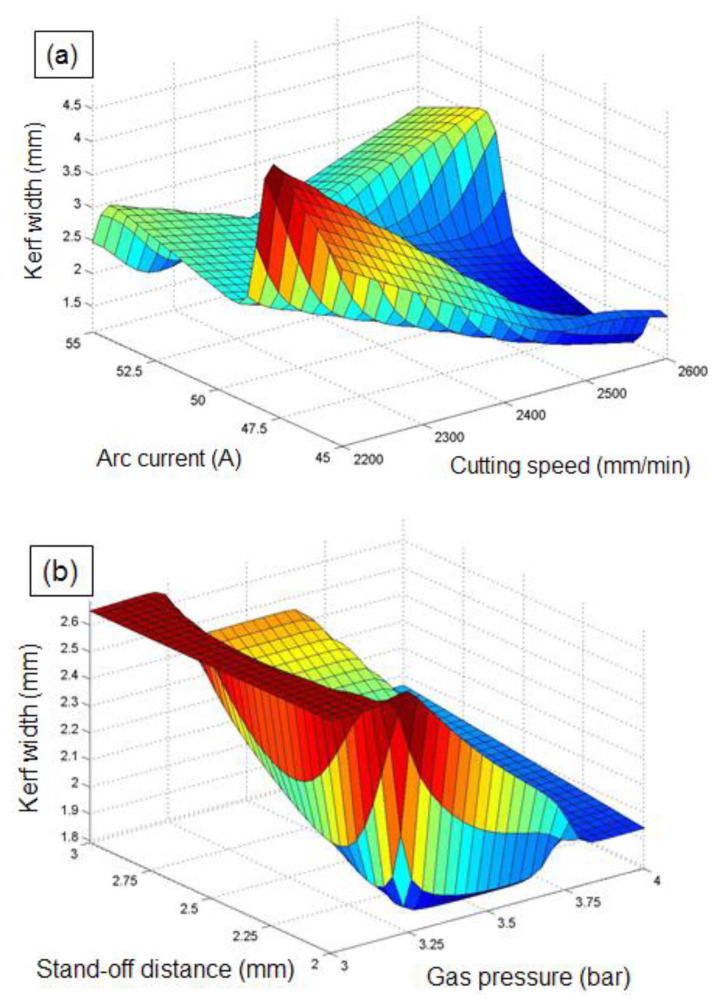
Influence of PAC parameters on *kw:* (**a**) influence of cutting speed and arc current on *kw*, and (**b**) influence of gas pressure and stand-off distance on *kw*.

The combined influence of PAC control parameters on the *kw* has been visualized with the aid of ANFIS 3D surface plots (Figure 12a,b). The possession of AC and CS on *kw* is depicted in Figure 12a. It can be seen that the increase in CS from 2200 mm/min to 2800 mm/min results in a decreasing trend in *kw*, whereas the increase in AC up to a certain level results in an augmented *kw* and then decreases with an increase in AC. High heat energy is established at the higher CS with lower AC to melt the material efficiently with adequate time; thus, lower *kw* is produced [61]. Moreover, due to the lower thickness of the substrate material, no obvious deviation in *kw* is observed at the cutting zone. 

The collective influences of SOD and GP on the *kw* is depicted in Figure 12b. As can be seen from the plot, the *kw* value decreases linearly with an increase in GP from 3 to 4 bar, whereas an increase in SOD from its lower to higher levels, along with GP, resulted in an augmented *kw*. At higher GP and arc current, the plasma jet expelled from the nozzle causes intense melting and vaporization, as well as an exothermic reaction, resulting in an irregular kerf width. This could be attributed to the fact that higher SOD facilitates an absence of arc coherence, resulting in arc swerving, which could increase vulnerability to peripheral drag from the surroundings of actual plasma. As a result, increasing the SOD results in an augmented plasma width and decreased kinetic energy at obtrusion, which leads to an improper cutting quality at the top and bottom kerf surfaces; hence the *kw* is increased [62]. An improved cut quality with a minimal *kw* of 1.8 mm is obtained at a combination of higher GP (4 bar) and lower SOD (2 mm).

**Figure 13:**
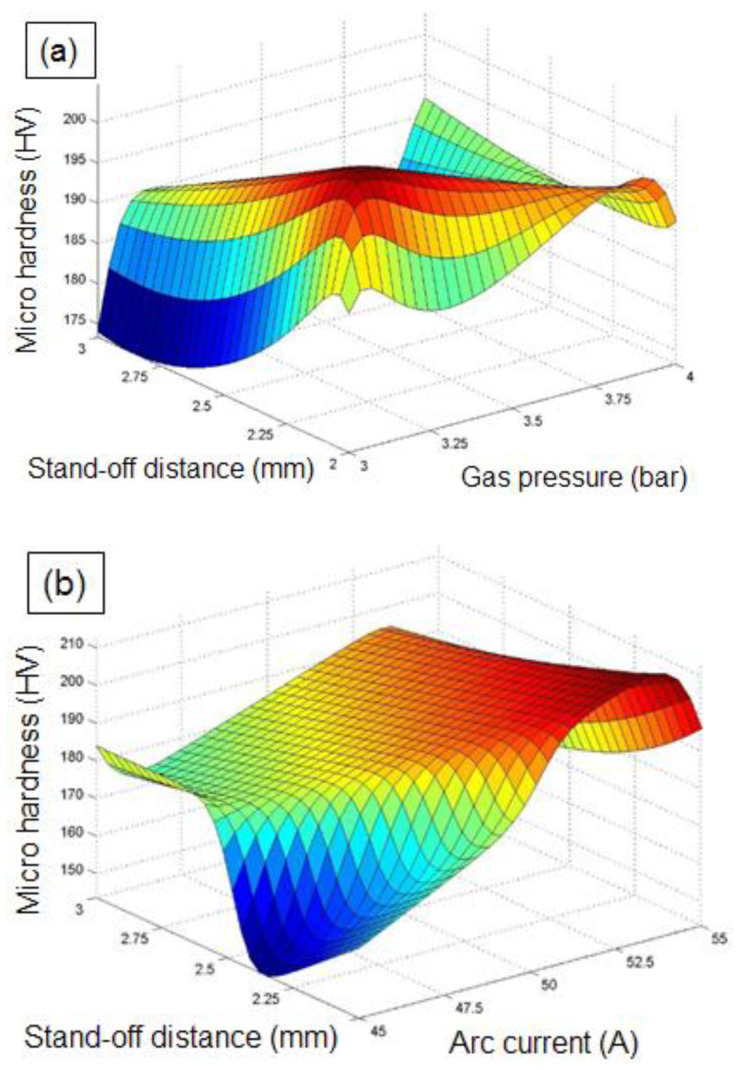
Influence of PAC parameters on *mh:* (**a**) influence of stand-off distance and gas pressure on *mh*, and (**b**) influence of arc current and stand-off distance on *mh*.

The influence of PAC control parameters on *mh* is described with the assistance of ANFIS three-dimensional plots (Figure 13a,b). The combinative influence of GP and SOD on the *mh* is presented in Figure 13a. The surface plots demonstrate that the *mh* increases with an increase in the parameters GP and SOD, from their lower to their higher values. The intensity of plasma expelled to the cutting zone will increase as the GP and SOD increase. As a result of the increased plasma thrusts, the recast layer at the kerf surfaces also progresses, as does the formation oxide layer at the cutting area; thus, the *mh* value increases [11]. Therefore, a suitable value of GP and SOD should be found for minimizing the micro hardness of cut surfaces.

Figure 13b demonstrates the effect of AC and SOD on the micro hardness of PAC processed Monel 400 sheets. The *mh* of the cut surface is found to increase with the intensification in AC and SOD. The heat energy transferred to the substrate surface increases as the AC and SOD intensifies from lower to higher values, which results in a higher heat affected zone around kerf surfaces. The increased heating zone results in amplified *mh* and oxide layer formation at the cutting zone [63]. From the plot, a minimized *mh* of 147 is observed at lower values of AC (45 A) and SOD (2 mm). 

### 4.4. Optimization of PAC Parameters through ABC Algorithm 

In this work, the objectives considered were the minimization of *Ra*, *kw*, and *mh*, which are the functions of the PAC control parameters, namely cutting speed, gas pressure, arc current, and stand-off distance. The optimized ANFIS multi-response models were converted as single objective functions using the TOPSIS statistical approach and were applied as the fitness functions for an artificial bee colony (ABC) algorithm. For the implementation of the proposed hybrid ANFIS-ABC approach, a computer code was developed in the MATLAB™ environment to integrate the FIS modeling and optimization algorithm. The ABC was initialized with the control parameters, as listed in Table 7, which were obtained from the existing works [64,65] to find the ideal combinations of optimal PAC parameters.

**Figure 14:**
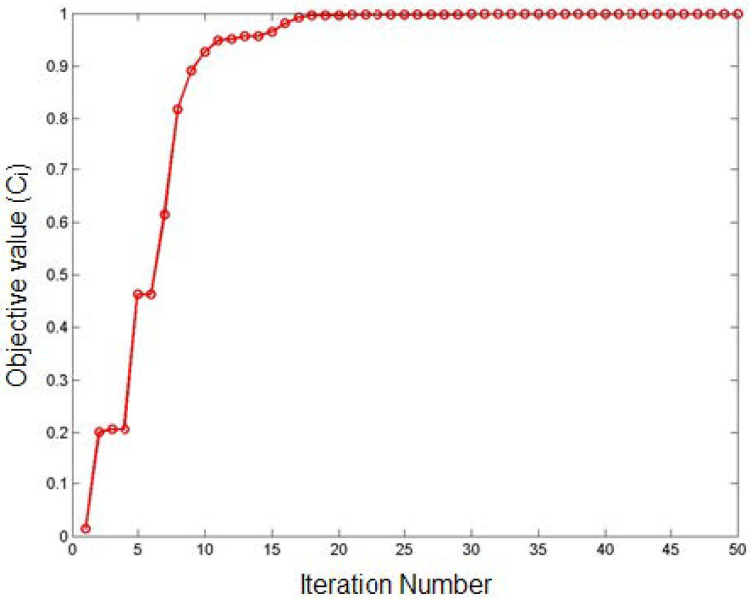
Performance of ABC Algorithm.

**Figure 15:**
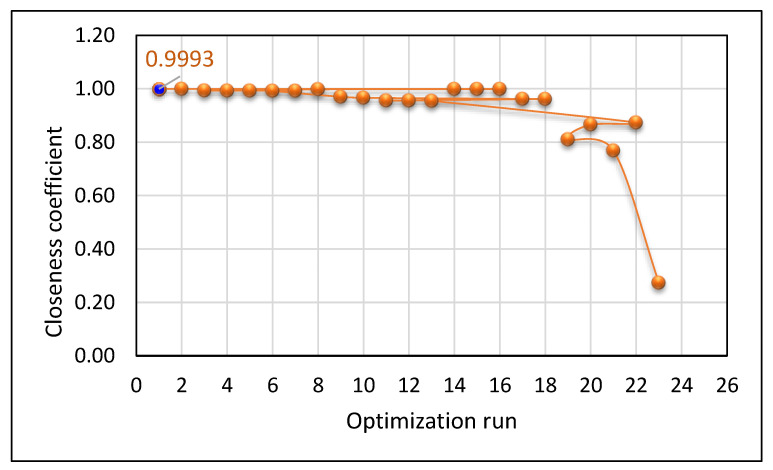
Graphical representation of obtained closeness coefficient values for each optimization run.

During the optimization using PAC, the parameters obtained at a higher closeness coefficient (objective function) is considered as the global best (optimal parameters) that satisfies the improvisation of *Ra*, *kw*, and *mh*. The proposed ABC was allowed to run for 50 iterations several times in order to achieve the optimal solutions. The performance of the ABC algorithm, which demonstrates the convergence of objective function with respect to the number of iterations, is shown in Figure 14. The algorithm was executed 23 times, and its corresponding response characteristic values were obtained (Table 8). The optimal PAC parameters for the improved quality characteristics of processed Monel 400 alloys were attained by statistically analyzing the obtained optimal parameter values through the TOPSIS approach. The higher closeness coefficient is considered for the present optimization problem, and their corresponding parameter values were considered as the global optimal parameters. The graphical representation of the obtained closeness coefficient for each optimization run is represented in Figure 15. From the statistical analysis, the following optimal PAC parameters were obtained at a closeness value of 0.9993, cutting speed of 2330.39 mm/min, gas pressure of 3.84 bar, arc current of 45 A, and stand-off distance of 2.01 mm. The corresponding response characteristics at the optimal cutting conditions are *Ra* of 1.5387 µm, *kw* of 1.2034 mm, and *mh* of 176.08.

In order to evaluate the rationality of the proposed optimization approach, a validation experiment was conducted based on the obtained optimal PAC parameters through the ABC algorithm. The results of the confirmatory experiments are listed in Table 9. It can be seen from Table 9 that the errors between the ABC predicted and the experimental values are 4.56% for *Ra*, 6.38% for *kw*, and 3.25% for *mh*. The confirmatory experiments show better agreement between the artificial bee colony algorithm predicted and the experimentally measured responses, with acceptable errors. Therefore, the proposed combined ANFIS-ABC can be suitable for the intelligent modelling and optimization of PAC parameters and their consequential response characteristics in order to improve the cutting quality. 

Conclusively, the findings of the proposed approaches indicate that the hybrid approaches of a parametric tuned GA-ANFIS and ABC can be effectively used for prediction modelling and the optimization of complex machining processes, namely, the plasma arc cutting process. The results of GA-ANFIS indicate that the prediction ability can be improved with minimized RMSE and MAPE values compared with the results obtained through MLRM. The hybridization of metaheuristic algorithms for tuning the ANFIS parameters is found to be efficient for reducing the computational capability with minimized experimental trials. By considering the satisfactory findings of the proposed hybrid approaches, similar techniques can be developed for intelligent modelling and optimization for improving the quality and performance characteristics of similar non-traditional machining processes.

## 5. Conclusions

The present work utilized a hybrid approach of ANFIS-ABC for intelligent modelling and multi-response optimization of PAC on Monel 400 alloy. The combined GA-ANFIS approach was utilized to generate models for forecasting the *Ra*, *kw*, and *mh* of processed alloy sheet. Multi-response optimization was performed for improving the cut quality of the PAC process using an artificial bee colony algorithm. From the preceding discussions of modeling and optimization studies, the following conclusions can be drawn:

- The accuracy of the proposed GA-ANFIS sub-clustering approach is enough to forecast the relationship between PAC parameters and response characteristics, with an average training and checking error of 0.058 and 0.299 for *Ra*, 0.022 and 0.132 for *kw*, and 0.797 and 2.741 for *mh*.
- The comparative evaluation of the proposed models indicated that the GA-ANFIS model is more efficient and exhibits a satisfactory enhancement in the forecasting of PAC parameters and their response characteristics, with minimal prediction errors such as RMSE and MAPE, compared with the MLRM approach.
- The ABC algorithm was found to be an efficient metaheuristic technique for optimizing the multi-response characteristics of the PAC process with fast convergence with fewer algorithm parameters. The obtained optimal parameters through the ABC algorithm are a cutting speed of 2330.39 mm/min, gas pressure of 3.84 bar, arc current of 45 A, and stand-off distance of 2.01 mm.
- The results of the confirmatory experiment show good agreement between predicted and experimental measured responses, having an error of 4.56% for *Ra*, 6.38% for *kw,* and 3.25% for *mh*. Therefore, it can be concluded that the ABC algorithm is an efficient technique for optimization studies in determining the optimal PAC process parameters.
- The efficiency of the proposed approach can be further utilized and enhanced by considering various parametric tuning algorithms, hybridization with other metaheuristics, and the processing conditions of various machining processes.

## Figures and Tables

**Table 1:** Selected PAC parameters and their levels.

Parameter	Symbol	Level			Units
		Low	Medium	High	
Cutting speed (CS)	A	2200	2400	2600	mm/min
Gas pressure (GP)	B	3	3.5	4	Bar
Arc current (AC)	C	45	50	55	A
Stand-off distance (SOD)	D	2	2.5	3	mm

**Table 2:** Experimentally measured response values.

Run	PAC Parameters				Surface Roughness (µm)	Kerf Width (mm)	Micro Hardness (HV)
	Cutting Speed (mm/min)	Gas Pressure (Bar)	Arc Current (A)	Stand-Off Distance (mm)			
1	2200	3.0	50	2.5	3.49	3.67	190
2	2600	3.0	50	2.5	2.70	2.75	164
3	2200	4.0	50	2.5	1.16	3.09	186
4	2600	4.0	50	2.5	3.43	2.49	189
5	2400	3.5	45	2.0	3.42	1.61	162
6	2400	3.5	55	2.0	2.46	2.22	196
7	2400	3.5	45	3.0	2.00	2.35	184
8	2400	3.5	55	3.0	1.65	2.13	170
9	2200	3.5	50	2.0	2.64	2.40	195
10	2600	3.5	50	2.0	4.26	2.34	184
11	2200	3.5	50	3.0	2.87	3.07	193
12	2600	3.5	50	3.0	2.79	1.97	177
13	2400	3.0	45	2.5	3.40	2.43	178
14	2400	4.0	45	2.5	1.76	2.54	154
15	2400	3.0	55	2.5	1.39	3.29	157
16	2400	4.0	55	2.5	1.77	2.27	202
17	2200	3.5	45	2.5	1.75	3.80	171
18	2600	3.5	45	2.5	2.49	1.74	163
19	2200	3.5	55	2.5	1.08	2.41	188
20	2600	3.5	55	2.5	1.64	3.18	161
21	2400	3.0	50	2.0	5.23	2.47	186
22	2400	4.0	50	2.0	1.81	1.94	191
23	2400	3.0	50	3.0	1.66	2.65	174
24	2400	4.0	50	3.0	3.77	2.06	192
25	2400	3.5	50	2.5	1.44	2.19	198
26	2400	3.5	50	2.5	1.48	2.11	197
27	2400	3.5	50	2.5	1.47	2.04	194
28	2400	3.5	50	2.5	1.41	2.15	193
29	2400	3.5	50	2.5	1.60	1.99	195
30	2400	3.5	50	2.5	1.78	2.02	192

**Table 3:** FIS parameters used for the investigation.

Parameters	Representation	Values/Range
RADII—Cluster radius	Four input parameters and a response (either *Ra* or *kw* or *mh*)	0.13 to 0.5
Quash factor	To multiply RADII values	2 to 3
% of data for training FIS model	Total number of experiments	65% to 85%

**Table 4:** GA parameters for optimizing ANFIS network.

GA Parameter	Value/Method
Population size	35
Method of selection for reproduction	Roulette wheel selection method
Crossover probability	0.4
Crossover operator	Single point crossover technique
Mutation probability	0.03
Mutation operator	Right side swapping
Replacement strategy	100% replacement strategy
Termination criteria	100

**Table 5:** ANFIS optimal parameters obtained through GA.

Response	RADII Value					% of Testing Data	Quash Factor	Training Error	Checking Error
	CS	GP	AC	SOD	R_a_/kw/mh				
*R_a_*	0.235	0.398	0.346	0.433	0.425	0.795	2.064	0.058	0.299
*kw*	0.456	0.270	0.269	0.316	0.259	0.761	2.646	0.022	0.132
*mh*	0.404	0.334	0.401	0.364	0.312	0.736	2.678	0.797	2.741

**Table 6:** Statistical validation results of proposed ANFIS-GA vs. MLRM.

Approach	MAPE			RMSE		
	*Ra*	*kw*	*mh*	*Ra*	*kw*	*mh*
GA-ANFIS	3.2289	1.6069	0.4350	0.1484	0.0668	1.5731
MLRM	4.9235	2.4279	0.7875	0.1716	0.0881	2.4278

**Table 7:** ABC algorithm parameters.

Parameters	Value
Bee’s position	A set of random values of parameters CS, GP, AC, and SOD within its lower and upper boundary values
Total number of bees	40
Number of Employed bees	20 (50% of total bees)
Number of Unemployed bees	20 (50% of total bees)
Selection of Onlooker Bee	Roulette wheel
Scout bees’ size	10 (50% of unemployed bees)
Stopping Criteria	50 iterations

**Table 8:** Optimal values obtained through artificial bee colony algorithm.

Run	Response Characteristics			Closeness Coefficient (*C_i_*)
	Surface Roughness (µm)	Kerf Width (mm)	Micro Hardness (HV)	
1	1.5387	1.2034	176.08	0.9993
2	1.5390	1.2027	176.21	0.9992
3	1.5406	1.2074	175.52	0.9931
4	1.5408	1.2069	175.59	0.9928
5	1.5410	1.2063	175.64	0.9926
6	1.5431	1.2062	175.57	0.9923
7	1.5431	1.2062	175.57	0.9923
8	1.5445	1.2795	162.97	0.9978
9	1.5566	1.2990	161.89	0.9698
10	1.5620	1.2913	162.84	0.9661
11	1.5625	1.2350	170.73	0.9559
12	1.5635	1.2355	170.74	0.9558
13	1.5691	1.2355	170.87	0.9545
14	1.5749	1.3629	159.31	0.9988
15	1.5749	1.3629	159.31	0.9988
16	1.5749	1.3629	159.31	0.9988
17	1.5795	1.2993	162.43	0.9619
18	1.5840	1.3195	161.60	0.9612
19	1.9250	1.9938	176.65	0.8102
20	1.9571	1.7901	176.50	0.8657
21	1.9743	1.7583	176.64	0.7682
22	2.1055	1.7068	179.23	0.8731
23	2.7206	1.6760	154.82	0.2733

**Table 9:** Validation results for *Ra*, *kw*, and *mh*.

Responses	Predicted	Experimental	Error %
Surface roughness (µm)	1.5387	1.6123	4.56
Kerf width (mm)	1.2034	1.2854	6.38
Micro hardness	176.08	182	3.25

## Data Availability

All the data are provided in the main text of the manaucript.
